# Optimizing Nutrient Uptake in the Critically Ill: Insights into Malabsorption Management

**DOI:** 10.2478/jccm-2024-0012

**Published:** 2024-01-30

**Authors:** Cristian Cobilinschi, Liliana Mirea

**Affiliations:** Carol Davila University of Medicine and Pharmacy, Bucharest, Romania; Anesthesiology and Intensive Care Clinic, Clinical Emergency Hospital of Bucharest, Romania

It has already been stated that nutritional support represents a crucial component in the care of critically ill patients [1]. Prolonged negative energy balance during intensive care stay was confirmed as an independent risk factor for mortality. High metabolic demand encountered for critically ill patients may cause significant energy deficits responsible for increased risk of infection, prolonged mechanical ventilation and ICU stay [2,3,4].

Additionally, providing nutritional support in ICU patients is often deemed challenging, as enteral feeding intolerance develops secondary to gastrointestinal dysfunction [5]. Excessive antimicrobial usage along with associated risk of nosocomial diarrhea may further exacerbate feeding intolerance.

Gastrointestinal dysfunction may be defined by a variety of functional impairments affecting motility, absorption, microbiome composition or perfusion, but still, at this moment there is a lack of recommendation regarding monitoring methods [6].

Central pathophysiological mechanisms implicated in critically ill gastrointestinal dysfunction are mostly related to gut oedema [6,7]. Multiple risk factors have been identified to contribute to the occurrence of gut oedema in ICU patients including systemic inflammation, associated capillary leak or inadequate fluid resuscitation [7,8,9,10,11]. Besides gut oedema, systemic inflammatory response related with surgical injury, infections, burns or toxics exposure may further promote endotoxemia by impairing intestinal motility [12,13].

At the bedside, assessment of gastrointestinal dysfunction often relies on measuring gastric residual volume (GRV) [14]. However, gastric emptying rate proved to be poorly correlated with GRV. This monitoring technique may also result in a decreased amount of nutrients delivery [15]. Since ultrasound has become a popular diagnostic tool also in the ICU, several studies indicated that ultrasonographic measurement of gastric antral cross-sectional area has a good correlation with both aspirated GRV and gastric volume measured by computerized tomography [16]. Considering that paracetamol has little to no absorption in the stomach and is completely absorbed at the intestinal level, paracetamol absorption test (PAT) has been proposed as a simple, indirect method for evaluating gastric emptying [17]. As the pharmacokinetic studies have established that gastric emptying is a rate-limiting step for paracetamol absorption, studies where PAT was used, validated a significant correlation with scintigraphy results [18].

Although it was thought that achieving optimal delivery of calories will prevent nutritional deficits in critically ill patients, published randomized controlled trials failed to confirm this hypothesis. Combining enteral and parenteral support may be an efficient strategy to reach nutritional target in critically ill patients. According to the current guidelines the use of supplemental parenteral nutrition (SPN) should be considered when energy targets are not achieved by enteral (EN) route, however, no clear data regarding timing, amount and composition is specified. Moreover, based on recent published data overfeeding should also be avoided, considering the negative impact on outcome.

Apart from discussing nutritional intake, great emphasis should be placed on nutritional uptake as an increased percentage of critically ill patients fail to reach nutritional targets often due to gastrointestinal dysfunction [16].

The combination of reduced tolerance to enteral feeding at the initiation of nutritional support, along with the initiation of nutritional support within 48 hours, has been proven to be responsible for a progressively significant and unmanageable energy deficit [9, 11]. It has been found that continuing enteral feeding under these circumstances is ineffective in resolving this energy debt which is proportional with an increased risk of nosocomial infections.

Although some authors opinioned that reducing energy targets might be beneficial, several studies proved that permissive underfeeding has no positive impact on outcome [19]. Moreover, reduction of energy target recommendation was associated with a secondary decrease of protein supplementation [20].

Using SPN for patients who cannot tolerate EN proved to be safe and was associated with improved cumulative energy balance, decreased rate of infections and significant cost reduction [21, 22]. Although SPN is proposed both by ESPEN and ASPEN guideline as an efficient alternative when energy and protein target are not achieved by oral or enteral route, recent data revealed that SPN use is rather limited [23]. The primary concern regarding SPN use is the risk of overfeeding. However, utilizing the appropriate concept of SPN and measuring energy needs by indirect calorimetry may overcome administering feeds in an unphysiological manner.

Besides intestinal absorption, efficient utilization of macronutrients should also be assessed, considering that critically ill patients have varying metabolic conditions and may not be able to metabolically handle administered substrates [24]. As a result, body composition analysis should be taken into account in order to obtain a dynamic quantification, especially, of the muscle mass compartments.

In addition to intake and uptake, muscle capacity to respond to nutritional protein should also be taken into account. Several different tools have been proposed for body composition analysis of critically ill patients [25]. Ultrasound with different protocols has also been used to assess muscle mass even in ICU patients with greater fluid shifts [25]. Studies have indicated that a significant reduction in muscle mass may be identified by both rectus femoris cross-sectional area and quadriceps muscle layer thickness measurements [25]. Bioelectrical impedance analysis is another non-invasive, low-cost technique used for body composition assessment. Despite reported limitations related with frequent overhydration states in critically ill patients, this method can still provide reliable data if the appropriate timing for examination is chosen [26]. Nevertheless, bioelectrical impedance analysis-derived phase angle proved to be a trustworthy parameter not only for evaluating fat-free mass, but also mortality [27]. Functional parameters, such as handgrip strength measurements should be also included when effectiveness of nutritional support is evaluated. However, it is important to acknowledge barriers to collecting functional outcome data particularly when critically ill patients are studied [23].

## Next in gastrointestinal dysfunction management?

Nutritional support for critically ill patients was focused more on preventing caloric and protein deficits and no great emphasis was placed on the efficiency of intestinal absorption. Gastrointestinal dysfunction is a prevalent reported complication that may contribute to falling short of meeting nutritional goals. This encompasses a wide spectrum of symptoms, such as impaired gastric emptying, ileus or impaired intestinal absorption, exposing patients to feeding intolerance, malnutrition and worse outcomes. No standard definition and monitoring techniques are so far available for the diagnostic of feeding intolerance. Although increased gastric residual volume (GRV) is the most used parameter for highlighting feeding intolerance, a controversy regarding the adequate threshold of GRV persists. Acetaminophen absorption test has been previously proposed as a diagnostic tool to asses impaired gastric emptying and intestinal absorption. Until recently, the paracetamol absorption test has been the most commonly employed method for assessing gastric emptying in critically ill individuals. This is because the area under the curve (AUC) is influenced by both the rate of gastric emptying and the absorption capacity of the small intestine [17]. However, there have been notable discrepancies in the applied protocols, encompassing variations in paracetamol dosage and form, as well as the type of meal with which it is administered. Additionally, there is a lack of uniformity in the calculated parameters, such as concentration at specific time points, maximal concentration, time to reach maximal concentration (*T_max_*), AUC, and the proportion of AUC at specific time points (*AUC_60/120/180_*) [28]. Nevertheless, this diagnostic tool seems to offer solid advantages and we consider that timely validation of this diagnostic tool in large prospective randomized trials is necessary to facilitate bedside diagnostic of critically ill patients with gastrointestinal dysfunction. This becomes more significant, considering that there is no other tool available for immediate use and all the tested markers such as enterohormones, acetylcholine or heparin binding protein are still not suitable for clinical use and the technology for their determination is more expensive [29].

In conclusion taking into account the impact of gastrointestinal dysfunction on the efficacy of nutritional support, we advocate that a settled monitoring technique is useful on the bedside in critically ill patients. Until then the question of whether if the doctor gives, the patients receive, is still unanswered.
